# Marked biological differences between insecticide resistant and susceptible strains of *Anopheles funestus* infected with the murine parasite *Plasmodium berghei*

**DOI:** 10.1186/1756-3305-6-184

**Published:** 2013-06-19

**Authors:** T Mike Lo, Maureen Coetzee

**Affiliations:** 1 Wits Research Institute for Malaria, School of Pathology, Faculty of Health Sciences, University of the Witwatersrand, Johannesburg, South Africa; 2 Division of the National Health Laboratory Service, Vector Control Reference Laboratory, Centre for Opportunistic, Tropical and Hospital Infections, National Institute for Communicable Diseases, Johannesburg, South Africa

## Abstract

**Background:**

*Anopheles funestus* is one of the major malaria vectors in Africa but research on this species has been restricted due to the lack of viable laboratory colonies. The vectorial capacity of natural populations of *An. funestus* is well known but its ability to host *Plasmodium* in the laboratory and the development cycle of the parasite within this mosquito species was, until very recently, unknown. In this study we compared laboratory strains of *An. funestus* that were resistant and susceptible to pyrethroid insecticides, for their receptiveness to infection with *Plasmodium berghei* and compared development times with other vector species available in our laboratory.

**Methods:**

The murine parasite *P. berghei* was used to infect two base *An. funestus* colonies (FANG and FUMOZ) and two selected sub-colonies with different degrees of pyrethroid resistance (FUMOZ-BS susceptible and FUMOZ-R resistant). Results were compared with the G3 strain of *An. gambiae.*

**Results:**

While all colonies were able to support the parasite, the development time in *An. funestus* was generally longer than that recorded in the laboratory strain of *An. gambiae*. Infected females were able to initiate new rounds of infection when feeding on healthy mice. The pyrethroid resistant strain FUMOZ-R supported the lowest numbers of oocysts and sporozoites while the insecticide susceptible strain FUMOZ-BS produced one of the highest sporozoite indices ever documented in *P. berghei* research. The FUMOZ base colony, exhibiting partial insecticide resistance was the median in terms of infection intensity. The oocyst number in all colonies did not fully correlate with the sporozoite index, indicating possible factors influencing the sporozoites’ transit from the midgut to the salivary glands.

**Conclusions:**

The presence of both insecticide resistance and limited parasite infection phenotypes in the same individuals suggests there may be association between the two mechanisms, but further elucidation is required.

## Background

Malaria remains the most serious tropical infectious disease, causing an estimated 660,000 deaths worldwide in 2010, of which 91% occurred in Sub-Saharan Africa [1]. The disease is caused by parasites of the *Plasmodium* genus that are transmitted to humans by anopheline mosquitoes. *Anopheles gambiae s.s.*, *An. coluzzii, An. arabiensis* and *An. funestus* are the four major vectors of malaria in Africa and the surrounding islands [2,3]. Of these four species, *An. funestus* has been the most difficult to rear in captivity and this has presented a major obstacle to understanding the interaction of this species with the *Plasmodium* parasite. The only successful attempt at colonizing the species beyond the F_10_ generation thus far is by Hunt *et al.*[4]. Two colonies, one originating from Angola and another from Mozambique, were established over ten years ago. A pyrethroid (permethrin) resistant sub-colony of the Mozambican strain was subsequently selected [4].

The murine malaria infection system is a popular alternative to *Plasmodium falciparum* for vector-parasite research. It is relatively inexpensive, easy to maintain and has similarities to the infection process of human malaria parasites [5]. Of the four known murine malaria parasites, *P. berghei* is the most popular choice for research [5]. There are only two previous accounts of attempts to infect *An. funestus* using *P. berghei*, one of them was unsuccessful [6]; probably because it was carried out before the impact of temperature on *P. berghei* sporogonic development was realized [7-9]. Furthermore, *An. funestus* is a highly anthropophilic mosquito, and its predilection for humans may have severely biased the feeding on mice. When the biology of *P. berghei* was fully understood, a number of mosquito species were found to be competent carriers for the murine parasite [10] and a very recent publication has shown that *An. funestus* is also a viable vector for *P. berghei* under the correct conditions [11]. This study used F-1 generation *An. funestus* from Mali, infected with a transgenic strain of *P. berghei* expressing green fluorescent protein and showed that *P. berghei* successfully completed its life cycle in this vector mosquito [11].

The present study aimed to determine the life cycle of *P. berghei* in laboratory strains of *An. funestus* that were selected for pyrethroid resistance and susceptibility.

## Methods

### Mosquito strains

The base *An. funestus* laboratory colony FUMOZ, originating from Mozambique and exhibiting low levels of pyrethroid resistance, was used to select two derivative colonies with different levels of resistance to permethrin: FUMOZ-R, resistant (selected previously) [4] and FUMOZ-BS, susceptible (derived in this study). FANG, a fully insecticide-susceptible strain originating from Angola [4] was used in the initial screening for infectivity rates but not for quantification of infections.

The permethrin susceptible FUMOZ-BS strain was selected as follows: 40 individual females were randomly chosen from the base FUMOZ colony and placed into separate egg-laying vials. The females were provided with a blood meal every 2–3 days until death. Egg batches were reared separately to adulthood. Half of the resulting adults from each egg batch were exposed to 4% permethrin for one hour and mortality recorded after 24 hours. Those batches giving >85% mortality were pooled together in a cage for mating. At the 10th generation the process was repeated to ensure susceptibility was maintained. All strains were maintained at standard insectary conditions of 25 ±2°C, 80 ±10% relative humidity, with 12 hours day/night cycle including 30 minutes transition dawn/dusk periods [4].

Seventy to ninety females were collected from each strain for each infection cohort and provided with a 10% sucrose solution for 10–14 days. To improve the rate of blood feeding, the sucrose solutions were removed 18–24 hours prior to their first infected blood meal.

The G3 strain of *An. gambiae* was used as a positive control for the infection process. The *An. gambiae* cohorts were maintained under the same conditions as *An. funestus*, but only 50–60 females were collected for each infection. Feeding of *An. gambiae* on infected mice occurred 3–4 days after eclosion as described above.

### Laboratory infection of mice with *P. berghei* and mosquito blood feeding

C57/Black strain mice were injected intra-peritoneally (IP) with 0.2-0.3 ml of *P. berghei* infected bloodstock with at least 10% parasitaemia [5] (ANKA strain; courtesy of R. Sinden, Imperial College; also obtained from MR4 resources). The mice were then maintained under standard conditions (20 ±1°C, 50% relative humidity) and provided with food and water. The infection in the mice was monitored using Giemsa stained blood smear slides [5]. A differential analysis was performed under light microscopy at 1000× magnification. At least 10 fields of view were counted per slide to calculate the percentage of infected red blood cells. Parasitaemia levels were recorded at least every alternate day from day 3 of the infection [5]. Three to four days after initial infection, 2–4 mice were anaesthetized and the starved mosquitoes allowed to feed for up to 30 minutes. The adults that did not take a blood meal were removed from the experiment. Thereafter, the fed mosquitoes were maintained at 20 ±1°C, 80 ±10% humidity. The females were refed with an uninfected blood meal 5–7 days after the first infected blood meal to enhance the infection.

Ethical clearance for the use of mice for *P. berghei* infection was obtained from the animal ethics committee of the National Institute for Communicable Diseases (ethical clearance number: 110/07, 2007).

### Monitoring of *P. berghei* infection in mosquito specimens

Female mosquitoes were dissected to assess the presence of oocysts and sporozoites in the midgut and salivary glands, respectively [5]. Midgut dissections began 8 days post infection (pi), while salivary gland dissections began 14 days pi. For each dissection, 10–15 females per cohort were used. The dissected midguts were suspended in phosphate buffered saline (PBS) on a glass slide under a coverslip. The midguts were examined for oocysts under 100-400× magnification, and the number of oocysts present were counted and recorded. The dissected salivary glands, also suspended in PBS on a glass slide under a coverslip, were ruptured to determine the presence of sporozoites (100-400× magnification). Where possible, the sporozoites were counted to quantify the degree of infection. The remainder of the females in cohorts with confirmed infection were allowed to feed on healthy mice to determine the infectivity of the sporozoites, and to complete the infection cycle [5]. The mice infected via the mosquitoes were maintained and monitored in the same manner as the IP-infected mice. Two-sample T-tests and one-way ANOVA were used to compare the feeding and infection rates between the strains, as well as to compare the oocyst and sporozoite numbers.

## Results

**Figure 1:**
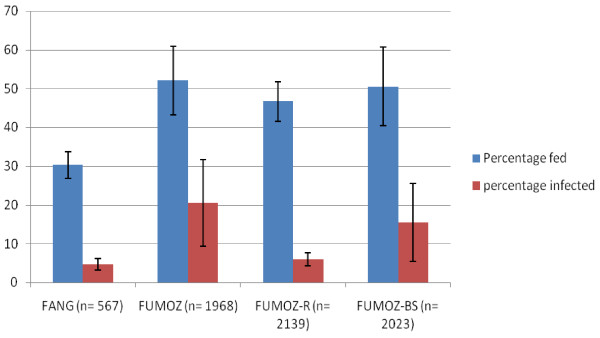
**Feeding and infection rates *of Anopheles funestus *colonies.**

Both *An. funestus* base colonies were susceptible to *P. berghei* infection, but at different rates (Table 1, Figure 1). FUMOZ mosquitoes exhibited a higher feeding success rate, with over 50% of the females (n = 1968) from 25 cohorts taking a blood meal, similar to the feeding rate seen in *An. gambiae* G3 strain where an average of 51.9% of the females took a blood meal. The insecticide susceptible FANG cohorts exhibited lower feeding success rate, with only 28-36% of the 567 females from 10 cohorts taking a blood meal. The FUMOZ cohorts also had higher infection rates. On average, 20% of fed females became infective, but some cohorts produced over 30% infection rates. Infection in FANG females was low and never exceeded 8% in any of the 10 cohorts. The *An. gambiae* G3 infection rates averaged 35.8%, ranging from 10.7 – 48.2%. The feeding and infection rates between FANG and FUMOZ were significantly different (p < 0.01). The feeding rates between FUMOZ and its derivative strains were not significantly different (p > 0.01) but the infection rates were (p < 0.01) (Figure 1).

**Table 1:** **Comparison of feeding and infection rates, as well as the average oocyst and sporozoite loads of *P. berghei *in *An. funestus *strains and other anopheline species**

**Colony/species (Total number of mosquitoes used)**	**Feeding rate (%)**	**Infection rate (%)**	**Average oocyst numbers (range)**	**Average sporozoite numbers (range)**	**References**
FANG (n = 567)	30.3	4.7	-	-	This study
FUMOZ (n = 1968)	52.1	20.5	24	1573	This study
FUMOZ-R (n = 2139)	46.7	6	6	1586	This study
FUMOZ-BS (n = 2023)	50.5	15.5	53	7249	This study
*An. dureni*	-	15.2	60-80	7400	Yoeli 1965 [10]
*An. quadrimaculatus*	-	42.5	100-500	100-300	Yoeli *et al.* 1965 [12]
*An. stephensi*	-	50.5	60-80	6840-8200	Yoeli *et al.* 1965 [12]
*An. funestus* trial 1	-	62.5	47 (1–141)	-	Xu *et al.* 2013 [11]
trial 2	-	95.0	120 (1–347)	-	

**Figure 2:**
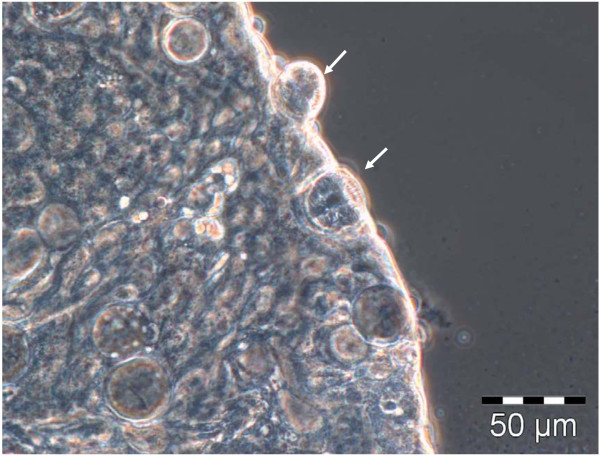
**Oocysts on *An. funestus *midgut at 20 days post infected blood meal.** The oocysts are at different stages of maturation, some containing immature sporozoites (white arrows) (phase contrast, 400 × magnification).

**Figure 3:**
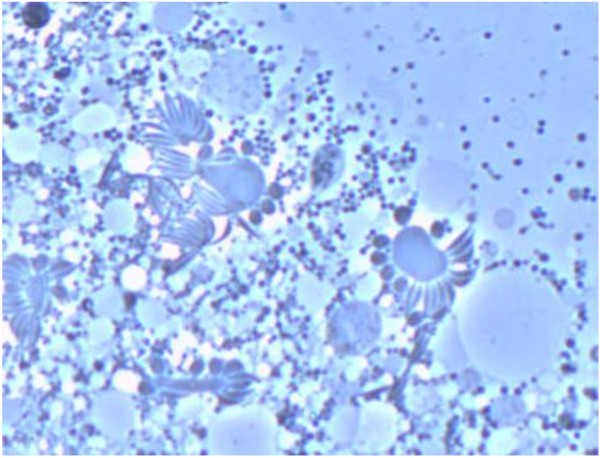
**Sporozoite assembly around blastophores, from ruptured oocysts in the midgut.** The sporozoites are all immature and at different stages of development (phase contrast, 400 × magnification).

Midgut dissections revealed oocysts 10 days pi at the earliest, with mature oocysts observed as late as 20 days pi (Figure 2). The average time for oocysts to appear was 14 days. Oocysts were not restricted to any specific region of the midgut, although they were more often located on the posterior half. Mature oocysts were 40 ±4 μm in diameter with sporozoites easily observed (Figures 2 and 3).

**Figure 4:**
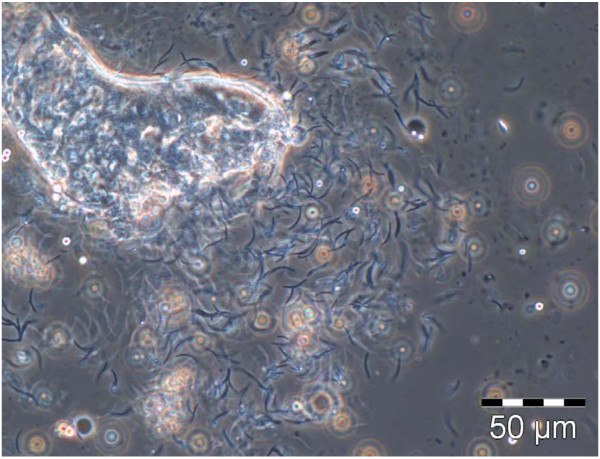
**Ruptured *An. funestus *salivary gland showing mature crescent sporozoites of *P. berghei *(phase contrast, 400 × magnification).**

Sporozoites were detected in the salivary glands as early as day 16 after the initial infected blood meal but this occurred in less than 1% of the infected females. The sporozoites were more consistently detected after 18–21 days of development in all colonies, and sometimes only appeared as late as day 24. The mature, infective sporozoites found in the salivary glands were 13 ± 2 μm in length, and had the classical crescent shape associated with malaria sporozoites (Figure 4).

**Figure 5:**
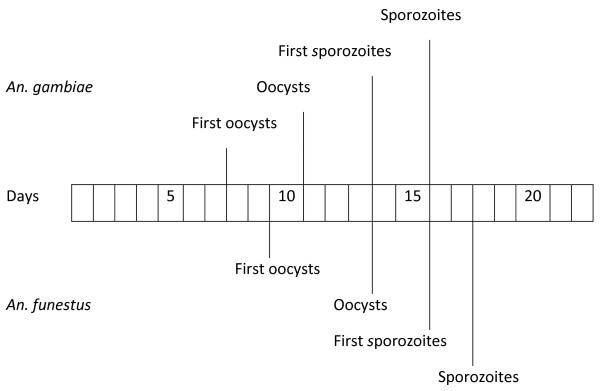
**A comparative timeline of *P. berghei *development in *An. gambiae *and *An. funestus.***

By comparison, the development of *P. berghei* in *An. gambiae* was faster under the same conditions, typically by 2–3 days. Oocysts were observed as early as 8 days, but on average appeared 11–12 days after infection. The earliest point when sporozoites were observed was on day 14, but was more consistently detected from day 16–20 (Figure 5).

Quantitative comparison of *P. berghei* developmental stages in the three FUMOZ strains (Tables 2 and 3) showed that FUMOZ-BS was most susceptible to *P. berghei*, being able to host at least 30 oocysts and carry on average over 7,000 sporozoites in its salivary glands. Oocyst and sporozoite numbers in FUMOZ-R were both much lower compared with the other two strains (Tables 2 and 3). A more detailed breakdown of the oocyst distribution showed that infected FUMOZ females carried a highly variable number of oocysts (ranging from 1 to 100), FUMOZ-R never supported more than 10 oocysts per female and over 60% FUMOZ-BS females contained more than 50 oocysts (Table 2). A similar breakdown of the sporozoite distribution showed an evenly distributed range of sporozoite numbers up to 10,000 in FUMOZ. In FUMOZ-R the number never exceeded 5,000 while FUMOZ-BS had 46.1% of the females with more than 10,000 sporozoites (Table 3).

**Table 2:** Comparison of oocyst load in 13–15 day post-infected females from the FUMOZ sub-strains

**Colony (number of infected mosquitoes dissected)**	**Average oocyst numbers (range)**	**Oocyst numbers**			
		**1 - 10**	**11 - 30**	**31 - 50**	**51 - 100**
FUMOZ (n = 59)	25 (1–60)	58%	10%	0	32%
FUMOZ-R (n = 64)	8 (1–15)	82%	18%	0	0
FUMOZ-BS (n = 65)	55 (27–83)	0	0	38.5%	61.5%

**Table 3:** Comparison of sporozoite load in 18–21 day post-infected females from the FUMOZ sub-strains

**Colony (number of infected mosquitoes dissected)**	**Average sporozoite numbers (range)**	**Sporozoite numbers**				
		**1 - 1 000**	**1 001–2 000**	**2 001–5 000**	**5 001–10 000**	**10 000–15 000**
FUMOZ (n = 59)	2248 (1075–3421)	20%	30%	30%	20%	0
FUMOZ-R (n = 64)	1586 (542–2630)	27%	53%	25%	0	0
FUMOZ-BS (n = 65)	7648 (2662–12634)	7.7%	15.4%	7.7%	23.1%	46.1%

Infection of healthy mice using infected *An. funestus* was carried out 21 days or later after an infected blood meal. Transmission of the parasite by *An. funestus* led to 84.2% of the mice becoming infected (n = 19), and all infected mice died within 9 days following cyclical transmission.

## Discussion

*Plasmodium berghei* is a popular alternative model organism in malaria research, but for over a decade after its discovery [13] there were only two isolated successes of infection in the laboratory [7]. *Anopheles funestus* was classified as a non-vector for the parasite during this time [6]. When lower temperatures were shown to be essential for *P. berghei* sporogonic development and invasion of the salivary glands [8-10], the vectorial capacity of *An. funestus* was not reassessed until very recently [11]. The lack of a laboratory colony and the highly anthropophilic nature of this species were possible factors contributing to its non-vector classification through the decades. However, the recent work carried out in Mali [11], and the current successful infection of *An. funestus* under the correct conditions indicate that this species is in fact a viable vector for *P. berghei*.

In the present study, the parasite displayed significantly different developmental times in *An. funestus* compared with other known *P. berghei* experimental vectors. The FUMOZ *An. funestus* females would only feed on mice with consistency 10–14 days after eclosion, significantly later than other anophelines (e.g. *An. quadrimaculatus* can be used for infection experiments 2–3 days after eclosion [7]) and twice the time recorded for the *An. funestus* from Mali [11]. The development time for the parasite was also delayed compared with other mosquitoes. In its natural vector, *An. dureni*, and the Asian malaria vector *An. stephensi, P. berghei* requires 10–11 days to complete sporogonic development [10,12], while *An. quadrimaculatus* is somewhat slower, requiring 13–14 days [14]. It is possible to observe sporozoites in *An. stephensi* salivary glands as early as nine days post-infection and on day ten for *An. quadrimaculatus*[8,9]. In *An. funestus*, the earliest salivary gland invasions were observed at 16 days post-infection, but sporozoites were more consistently seen in the salivary glands 18–21 days after infection. This is consistent with results for *An. funestus* from Mali [11].

Once the sporozoites had successfully invaded the salivary glands, the female *An. funestus* carried sporozoites in the salivary glands for the remainder of her life. Successful infection of healthy mice was achieved using infected *An. funestus* females 60 days after eclosion.

Both unselected *An. funestus* colonies, FANG and FUMOZ, were susceptible to *P. berghei* infection. However, FANG had both a low feeding and infection rate, making it less useful for experimental purposes. FUMOZ was thus the strain of choice for establishing the infection system. In an effort to resolve whether the partial insecticide resistance phenotype of FUMOZ had an impact on its vectorial capacity, two sub-colonies with varying permethrin resistance were also tested for infectivity. All three colonies had similar feeding rates but the infection rate was highly variable. FUMOZ-R, exhibiting high levels of permethrin resistance [4], was the most resistant to parasite infection, with low oocyst and sporozoite counts. Although the oocyst load in FUMOZ-R was amongst the lowest detected in experimental vectors [7,10,12], considerable numbers of sporozoites were still able to migrate to the salivary glands, thus maintaining its viability for parasite infection. FUMOZ-BS, selected for the pyrethroid susceptible phenotype, was highly susceptible to *P. berghei* infection, typically carrying a modest oocyst load. Although the oocyst load in FUMOZ-BS is not high compared with the frequently used experimental vector *An. stephensi*, the sporozoite index in the *An. funestus* strain is amongst the highest recorded using the murine parasite [10,15]. The FUMOZ-BS sporozoite load in this study surpassed that observed in *P. berghei*’s natural vector, *An. dureni*[10]. The unselected FUMOZ base strain was the median, with fairly light oocyst growth and highly variable sporozoite numbers.

Although oocyst load in *An. funestus* usually corresponded with sporozoite index, it was not an absolute correlation and some discrepancies were observed where low oocyst numbers (<10) led to heavy sporozoite infections (>2,000) as in the case of FUMOZ-R. Conversely, the sporozoite numbers observed in FUMOZ were sometimes lower than expected given that 32% of the females had >50 oocysts present. It is unclear what factors affect the transit of the sporozoites between the midgut and salivary glands and this will require further investigation.

The presence of resistance to insecticide and low parasite infectivity in the FUMOZ-R (and conversely, susceptibility to both factors in FUMOZ-BS) suggests that there may be some association between these two phenotypes. There are currently no known genes or pathways that act in both insecticide resistance and parasite infection in anophelines. The most comparable model is *Culex quinquefasciatus* and the lymphatic filariasis parasite, *Wuchereria bancrofti*. Insecticide resistant *Cx. quinquefasciatus* exhibiting increased esterase activity, affects the development of *W. bancrofti* larvae possibly by arresting the parasites in the gut cells [16]. The correlation was easier to quantify in *Cx. quinquefasciatus* as the esterase amplicon duplication is the dominant resistant mechanism, found in over 80% of insecticide resistant *Cx. quinquefasciatus*[17]. The phenomenon may be more difficult to correlate between the two phenotypes in anophelines, since malaria vectors employ a number of insecticide resistance mechanisms [18-22] that are involved in different manners depending on the type of insecticide the mosquitoes are exposed to. However, the pyrethroid resistance in southern African *An. funestus* has been well-studied showing that mono-oxygenase detoxifying enzymes in the CYP6 class are mainly responsible [23-27]. It is possible that these enzymes are preventing *P. berghei* parasite development in *An. funestus* similar to the esterase enzymes in *Cx. quinquefasciatus* inhibiting *W. bancrofti* development.

## Conclusions

The results indicate that metabolic insecticide resistance plays a role in determining the infectivity of *An. funestus* to *P. berghei* parasites with resistant strains being less condusive to parasite development than susceptible strains. Further investigations are needed to determine whether infectivity of the vectors is influenced by their insecticide resistance profile in natural populations with respect to *Plasmodium falciparum* as this may have implications for future vector control interventions.

## Competing interests

The authors declare that they have no competing interests.

## Authors’ contributions

TML carried out the experiments and drafted the manuscript. MC conceived the project and assisted with the drafting of the manuscript. Both authors read and approved the final version of the manuscript.
